# The relations between growth mindset, motivational beliefs, and career interest in math intensive fields in informal STEM youth programs

**DOI:** 10.1371/journal.pone.0294276

**Published:** 2024-04-09

**Authors:** Emine Ozturk, Mengya Zhao, Angelina Joy, Christina S. Marlow, Fidelia Law, Ashley R. Deutsch, Channing J. Mathews, Adam J. Hoffman, Luke McGuire, Mark Winterbottom, Frances Balkwill, Karen Burns, Laurence Butler, Marc Drews, Grace Fields, Hannah Smith, Adam Rutland, Adam Hartstone-Rose, Kelly Lynn Mulvey

**Affiliations:** 1 Department of Psychology, North Carolina State University, Raleigh, North Carolina, United States of America; 2 Department of Psychology, University of Exeter, Exeter, United Kingdom; 3 Department of Primary Care and Mental Health, University of Liverpool, Liverpool, United Kingdom; 4 Department of Human Development and Family Science, Purdue University, West Lafayette, Indiana, United States of America; 5 School of Education, University of Bristol, Bristol, United Kingdom; 6 Department of Biological Sciences, North Carolina State University, Raleigh, North Carolina, United States of America; 7 Department of Psychology, University of Virginia, Charlottesville, Virginia, United States of America; 8 Department of Psychology, Cornell University, Ithaca, New York, United States of America; 9 Faculty of Education, University of Cambridge, Cambridge, United Kingdom; 10 Centre of Cell, Queen Mary University of London, London, United Kingdom; 11 Virginia Aquarium & Marine Science Center, Virginia Beach, Virginia, United States of America; 12 Thinktank Birmingham Science Museum, Birmingham, United Kingdom; 13 EdVenture Children’s Museum, Columbia, South Carolina, United States of America; 14 School District Five of Lexington and Richland Counties, Columbia, South Carolina, United States of America; 15 Florence Nightingale Museum, London, United Kingdom; University of Dhaka, BANGLADESH

## Abstract

Past research has shown that growth mindset and motivational beliefs have an important role in math and science career interest in adolescence. Drawing on situated expectancy-value theory (SEVT), this study extends these findings by investigating the role of parental motivational beliefs (e.g., expectancy beliefs, utility values) and parent growth mindset in math on adolescent career interest in math-intensive fields (e.g., mathematics, computer science, statistics, and engineering; MCSE) through adolescent motivational beliefs in math. Structural equation modeling was used to test the hypothesized model using data from 290 adolescents (201 girls, 69.3%; *M*_*age*_ = 15.20), who participate in informal STEM (science, technology, engineering, mathematics) youth programs, and their parents (162 parents, 87.7% female) in the United Kingdom and the United States. As hypothesized, adolescent expectancy beliefs, utility values, and growth mindset in math had a significant direct effect on MCSE career interest. Further, there was a significant indirect effect of parental expectancy beliefs in math on MCSE career interest through adolescents’ expectancy beliefs. Similarly, there was a significant indirect effect from parental utility values in math to MCSE career interest through adolescents’ utility values. The findings suggest that parents’ math motivational beliefs play a critical role in adolescent math motivational beliefs and their career interest in math-intensive fields.

## Introduction

Despite efforts to bolster enrollment in science, technology, engineering, and mathematics (STEM) fields to support expected growth in related industries, there is a deficit of graduates in these fields at the global level [1]. In two recent reports [2,3], the skills shortage in STEM fields has been accentuated as a major problem for economic growth that requires continued urgent action at the national level. Moreover, students’ math achievement is at the lowest levels in the last twenty years [4] and the attrition rate from math intensive careers, in particular engineering, is the highest compared to all other majors in the last few decades [5–7]. A robust STEM workforce is necessary to promote economic resilience and future prosperity [8]. This may be especially important for math-intensive careers where the need is quite high: the expected growth rate for computer science and mathematical occupations is 15.2%, which is the second highest rate among all other occupations in the US, after healthcare support occupations in the US [9].

Extensive research has shown that there is still an inequity in the representation of women and, ethnic and racial minority individuals in certain STEM fields like math, computer science, and engineering [2,10–13]. Career interest is a major determinant of individuals’ choice of a career [14,15]; therefore, increasing interest and attractiveness of math and science careers among youth at all levels of formal education is a high-level priority [3,16].

Research suggests that promoting interest in careers in math-intensive fields might play a critical role in closing the skill and gender gap, pay disparities, and economic inequities, especially for women and racial and ethnic minorities [17,18]. STEM is a broad categorization capturing a range of academic and career domains [19], and research suggests that children and adolescents do not hold the same stereotypes and conceptions of each domain within STEM [20]. Further, there is a need to encourage interest prior to college to ensure that adolescents are well-positioned for entry into the workforce [21].

Although studies have recognized the importance of self-perceived abilities and beliefs in STEM career interests [22–24], the role of parent factors such as parent mindset [25] and parental motivational beliefs [26] on adolescent motivation has been largely unexamined [27]. Moreover, there is still much unknown about the associations between parent and adolescent mindsets [28]. This study examines the relationships between adolescent and parent growth mindset, their expectancy beliefs and utility values in math, and adolescents’ career interest in math-intensive fields (e.g., math, computer science, statistics, and engineering; MCSE).

### Informal STEM youth learning programs

Although much of the prior research has focused on factors that motivate STEM career interests with attention to learning in formal settings [29–31], some adolescents also have the opportunity to engage with STEM in out-of-school contexts, for instance through afterschool programs at museums, zoos, and aquariums [32,33]. In fact, prior research documents that informal STEM learning experiences can promote STEM career interests [34–36]. Informal STEM learning programs may be especially important for promoting career interest for students who are typically marginalized in STEM fields, as these programs can promote feelings of belonging [37] and inclusion as well as interest in STEM [36]. However, prior research on adolescents involved in informal STEM programs has not focused on math outcomes specifically. Research suggests that math-related constructs predicted STEM trajectories and career choices [38]. Given that little research has focused on math outcomes for those involved in informal STEM program, there is a high demand for this type of program [39]. These programs often focused on increasing STEM career interests [33], and there is a need to understand what factors shape MCSE career interest for adolescents involved in these types of programs. Therefore, the current study aims to understand relationships between parental and adolescent shared motivational beliefs, math growth mindset, and adolescents’ MCSE career interests in a sample of participants from informal STEM youth programs from 10 to 20 years of age.

### Theoretical framework

Expectancy-value theory [EVT; 40–42] has provided evidence supporting the crucial role of motivational and social factors including ability self-concepts, beliefs, values, and intellectual competencies in math career interest and aspirations [24,43–45]. Eccles [40]’s theoretical framework considers two motivational constructs: expectancy beliefs and subjective task values. Expectancy beliefs and utility values are two critical precursors for math-related career plans, achievement goals, vocational choices [46]. In this study, we focused on expectancy beliefs and utility values in math domain. Expectancy beliefs are conceptualized as individuals’ task-specific expectations for success, self-confidence, and ability beliefs in a particular domain [41,42]. Subjective task value refers to perceptions about the values in a particular domain and includes four subcomponents: intrinsic value, attainment value, utility value and perceived cost [40,42]. Utility value is defined as perceptions about the usefulness of a particular task [40].

Recently, Eccles and Wigfield [47] extended EVT as *situated* expectancy-value theory (SEVT) and highlighted the importance of considering contextual influences, culturally bounded and situated expectancies and values, and the extensive role of proximal socializers (i.e., parents’ general and domain-specific expectancies and values). According to SEVT [47] and the parental socialization model [48], parents’ general, specific, and gender-typed beliefs and values may impact their children’s career orientations. For example, Bleeker and Jacobs [49] examined the longitudinal associations between mothers’ earlier perceptions of their adolescents’ math ability beliefs, adolescents’ self-perceived math ability and their math career choices. The study revealed that mothers’ perceptions were significantly related to their children’s perceived abilities in math and later math career choices. SEVT provides a basic theoretical framework and broad guidance on the situational nature of informal STEM youth learning programs, and general and domain-specific family socialization processes on shared motivational beliefs and outcomes in this study.

### Does adolescent’s mindset matter?

A growth mindset is the implicit belief that intellectual abilities are malleable and can be improved through practice; a fixed mindset is the belief that intellectual abilities are static and fixed [50,51]. Mindset theory (implicit theories of intelligence) provides useful insights into how ability beliefs influence motivation and interest, with findings documenting that math mindsets are important in shaping math motivation and interest [52]. Students who believe math ability is an innate ability might be disadvantaged (e.g., low level of persistence and effort in math tasks) compared to students who believe math is a learned ability [52,53].

It has been demonstrated that growth mindset and motivational beliefs are positively associated [43,54,55]. A growth mindset can improve students’ motivation and persistence through challenge seeking and willingness to persist in difficult learning experiences [56]. However, previous research demonstrated inconsistent findings in the relationship between domain specific growth mindset and particular components of motivational beliefs [22,44,57]. Degol and her colleagues [22] examined associations between math growth mindset, expectancy beliefs, subjective task value, and STEM career aspirations high school students from the U.S. Though their findings indicate a significant association between math growth mindset and subjective task value, the association between math growth mindset and expectancy beliefs was not significant. Dowdy [57] also reported that the relationship between math mindset and math motivation was not statistically significant in a sample of ninth-grade at-risk students who were enrolled in an alternative school in math in the U.S. Heyder et al. [44] examined the role of math fixed mindset on motivation in math and language arts German high school students. Their findings indicate that there is a significant association between math students’ fixed mindset and their math motivational beliefs only for females but not for males. However, it is not clear what parent factors (e.g., parent mindset) in math may be important in the relationship between math mindset, motivational beliefs (e.g., expectancies for success and perceptions of utility) and math career orientation in adolescence.

### Parent mindset and motivational beliefs predictors of adolescent’s motivational beliefs

Although studies have recognized the impact of parent factors on student motivation [26,58,59], far too little attention has been paid to the role of parent mindset on academic outcomes in adolescence. Much of the literature concerns teacher mindset in school and classrooms [51,60,61] rather than mindsets in informal learning settings [25]. Cheng et al. [28] examined the mediating role of parental mindset on the relationship between student mindset and STEM outcomes. Results revealed that parental math growth mindset was associated with students’ math growth mindset for girls; however, parental math mindset did not provide benefits to girls in developing occupational interest in engineering and computer sciences. Recent research has tended to show parents’ fixed math mindset was negatively associated with students’ motivation in math [25]. However, it is unclear to what extent parental growth mindset is associated with parents’ and adolescents’ shared motivational beliefs and academic outcomes in MCSE fields.

Parents have significant influences on their children’s motivational beliefs and values [58,59,62–64]. For example, Tiedemann [64] examined the relationship between parents’ beliefs about their children and their child’s perceived ability in math third and fourth grade children in Germany. Results indicated significant associations between parents’ specific beliefs about their child and their child’s ability beliefs in math. Indeed, parents’ domain-specific beliefs and utility values in math might have a critical influence on their children’s self-perceptions and utility values in this particular domain [63].

Using EVT, Harackiewicz et al. [63] developed an intervention in to test how parents’ utility values affect the students’ academic motivation in math tenth and eleventh grade students and their parents in the U.S. They reported that parental utility value had a significant impact on their children’s math and science motivation and academic interest in high school years. Therefore, in the current study, we focus on exploring the role of parent math growth mindset and motivational beliefs, in particular.

### Links between growth mindset and motivational beliefs to MCSE career interest

Mindsets are associated with math career orientation [53]. Students with fixed math mindset might perceive themselves as less capable which might be a leading reason why they avoid math-intensive careers [52]. For instance, Degol et al. [22] examined the role of expectancy beliefs and task utility values as mediators in the relationship between student math growth mindset, and their STEM career aspirations. Although, the relationship between expectancy beliefs, adolescent growth mindset, and STEM career aspirations was not statistically significant, they did establish a link between task utility value, growth mindset, and STEM career aspirations.

Motivational beliefs and values are important factors in student career interests and aspirations [42,65–68] in particular for engineering and computer science career interest [69]. Students who feel confident about their abilities in math and science are more likely to pursue a career in these fields [24,40]. Using a longitudinal approach, Wang [24] examined the associations between student math motivational beliefs (i.e., expectancies and subjective task values) and career aspirations in math related areas from sixth grade (baseline) to twelfth grade (final wave) in the U.S. Results revealed that there were significant associations between student expectancies, subjective task utility values in math and career interest in math intensive fields. Collectively, these studies suggest that adolescents’ motivational beliefs might play a critical role in the association between math growth mindset and math-intensive career interest.

### Links between parents’ and adolescents’ motivational beliefs and MCSE career interest

Parental expectations and perceived importance might shape adolescents’ expectations and values directly and indirectly in a particular domain [49,70,71]. Using a longitudinal approach, Simpkins and her colleagues [72] examined the associations between mothers and their children’s expectancy beliefs in math. They reported that a positive indirect effect between mothers’ and youth expectancy beliefs in math through parent behaviors (e.g., role modeling, daily co-activity, encouragement). In another major study, Bleeker and Jacobs [49] found that mothers’ perceptions of their children’s math ability in middle school predicted their children’s perceived math ability in 10^th^ grade and later math career choices. Other studies have concluded that parents’ high math-related expectancy beliefs are negatively associated with adolescents’ math expectancy beliefs [73]. Wang et al. [73] found that students with no specific math-achievement expectations from their parents reported more positive math expectancy beliefs compared to students who perceive math-related expectations from their parents in 10^th^, 11^th,^ and 12^th^ grade.

There is a large number of published studies [e.g., 26, 74–76] that describe the association between parental and adolescent utility values. Jodl et al. [77] found that there is a direct effect of parental values on adolescent values in math. Gniewosz and Noack [74] make a similar point in their study of the intergenerational transmission of math values within families. They found that both mothers’ and fathers’ valuing of math were significant predictors of students’ own values in math; however, mothers’ impact on students’ values is larger compared to fathers’ impact in the math domain. The evidence reviewed here seems to suggest a critical role of parental motivational influence on adolescents’ motivational beliefs.

Previous research has established that students’ career choices might be shaped by the shared motivational beliefs of parents and adolescents [26,65,77,78]. Jodl and her colleagues [77] found that parental values predicted directly and indirectly adolescents’ career aspirations. Degol et al. [22] reported that adolescent math utility value predicted STEM career aspirations, but math expectancy beliefs did not predict their career aspirations in STEM.

These studies clearly indicate that there is a relationship between parental and adolescent shared motivational beliefs in math; however, very little is known about the extent to which adolescents’ motivational beliefs influence the association between parental math motivational beliefs and adolescents’ math intensive career interest in an informal learning context. To better understand the relationship between shared motivational beliefs of parents and adolescents, and math intensive career interest, we tested the direct and indirect effects of parent factors in this study.

### The importance of adolescence

Adolescence is a key developmental period when youth are developing an interest in pursuing math and science careers [49]. Students with low math motivation might avoid math-related careers in this transitional phase. Research has shown that career goals and interests become more realistic and relatively stable in early adolescence [79]. Many career-related decisions have been made in family context [80]; therefore, parents’ perceptions and beliefs play a key role in career interests and choices for adolescents [81]. Although a considerable body of research focused on parent factors [25,82,83], less attention has been paid to which parent factors influence adolescents career intentions in math-intensive fields in particular [84]. Recent research [38] revealed that the direction of the actual or perceived parent factors on adolescents’ motivational beliefs in particular STEM fields is not clear. Watt et al. [38] argue that adolescents’ perceptions of mothers’ math ability beliefs might have a greater impact on adolescents’ motivation compared to parents’ self-reported math motivational beliefs. With respect to parent mindset, Haimovitz and Dweck [85] reported that there was no significant correlation between parents’ and adolescents’ self-reported mindsets. These results might suggest that there is a difference between parents’ self-reported mindsets and adolescents’ perceptions of their parents’ mindsets [25]. However, adolescents’ self-perceptions are also shaped by their parents’ interpretations [26]. Therefore, this study set out to clarify the role of parent factors on adolescents’ motivational beliefs and their career interests in MCSE.

### Current study

This study offers new approach model for understanding pathways to math intensive fields for adolescents participating in informal STEM youth programs in the U.S. and the U.K., two countries in which persistent skill shortages in MCSE have been of systemic concern [2,3]. The aim of the current study is to explore the associations between adolescent and parent growth mindset, shared motivational beliefs and adolescent career interest in MCSE. Additionally, this study aims to explore the indirect effects of parent factors (i.e., growth mindset and motivational beliefs) on adolescents’ math-intensive careers through adolescents’ motivational beliefs.

Using SEVT as a guiding framework, and the above-described previous findings, the present study explores the following research questions: (1) Does adolescent growth mindset predict their motivational beliefs in math (i.e., expectancy beliefs and utility values) and MCSE career interest? (2) Does parent growth mindset predict adolescent motivational beliefs in math and MCSE career interest? (3) Do parental motivational beliefs (expectancy beliefs and utility value) predict adolescent motivational beliefs in math and MCSE career interest? (4) Is there an indirect effect of parental motivational beliefs on adolescents’ MCSE career interests via adolescents’ motivational beliefs? (5) Is there an indirect effect of parent growth mindset on adolescents’ MCSE career interests via adolescents’ motivational beliefs? We predicted that parent and adolescent math growth mindset, as well as parental expectancy beliefs and utility values in math would be positively related to adolescents’ expectancy beliefs, utility values in math and MCSE career interests (See hypothesized model). We also hypothesized that parent math growth mindset would predict MCSE career interest via adolescents’ motivational beliefs in math. Further, we expected that parental motivational beliefs would predict MCSE career interest via adolescents’ motivational beliefs in math.

## Methods

### Participants

Data were drawn from a larger ongoing longitudinal research project on informal STEM youth programs in the U.S. and U.K. Participants in this study consisted of 290 adolescents (69.3% girls; *M*_*age*_ = 15.20; *SD =* 1.65; 45.9% White, 23.1% Asian, 13.8% Black, 7.2% dual heritage, 2.1% Native Hawaiian/ American Indian/Hispanic, 7.9% Other) and their parents. The participants were recruited from six informal STEM youth programs in the U.K. and the U.S. The U.K. sites include a science museum (10.0% of participants), a science education center (30.7%), biomedical history museum (3.1%). The U.S. sites included a zoo (15.2%), a children museum (7.6%), and an aquarium (33.1%). The sites in the U.K. and the U.S. have similar structures and goals regarding student science experiences, engagement, and learning, with all of the programs, focused on providing adolescents with STEM knowledge and giving them the experience of serving as youth educators in the site and sharing what they learned with the visitors to these sites [37]. Parents were invited to complete one questionnaire when their adolescents enrolled in the informal STEM learning programs. Forty-four percent (*n* = 162) of parents agreed to participate. Of the 162 parent participants, 87.7% were mothers. This investigation focused on adolescents’ responses prior to starting the informal STEM youth program (baseline data collected before beginning the program). A priori power analysis was conducted [86] to estimate the required sample size for the hypothesized SEM model. The results showed that a total sample of 288 participants was required to detect small effects (*d* = 0.20) with power of 0.80, and an alpha of 0.05.

### Procedure

This study received approval by the Human Research Ethics Committee of Goldsmiths, University of London in the U.K. and the Institutional Review Board at North Carolina State University in the U.S. (Ethics approval number is 21017). The principal investigators received an inter-institutional agreement at first and then fully renewed it at North Carolina State University. Parents of potential participants from six informal STEM youth programs were asked for opt-out consent to participate and their permission for the participation of their adolescents. Prior to commencing the study, opt-out informed consent forms were sent via email to the parents of potential participants in the U.S. Parents of potential participants who were under 16 had to opt-in for the participation of their child in the U.K. Participants over 16 were eligible to give their own consent in the U.K. We sent an email invitation to all potential participants who had parental consent and did not need consent based on their age. Parents were notified of the study with information about the purposes of the study, confidentiality, and voluntary participation. The recruitment period started May 2017 and the questionnaires were sent to the adolescents and their parents who agreed to participate via Qualtrics software. Adolescents who completed the survey were compensated with a small electronic gift card. Parents did not receive compensation for participation. In the data preparation phase of the study some coauthors had the ability to directly identify participants during the data collection. This was necessary in order to match their names to their participant identification at each time point. However, all direct identifiers were removed before analyses.

### Measures

#### Math growth mindset

Parent growth mindset (PGM) and adolescent’s growth mindset (AGM) in math were assessed with the following item: “Most people can learn to be good at math” [87]. The response of scale item ranges from 1 (*strongly disagree)* to 7 (*strongly agree)*, where a higher score represents a greater growth mindset in math.

#### Adolescents’ Math Expectancy Beliefs (AEB)

Five items were used to measure adolescents’ expectancy beliefs (AEB) in math [29]: (a) “How good are you at math?” (1 = *not at all good*; 7 = *very good*); (b) “If you were to list all the students from best to worst in math, where are you?” (1 = *the worst*; 7 = *the best*); (c) “Compared to other subjects, how good are you at math?” (1 = *not at all good*; 7 = *very good*); (d) “How well do you expect to do in math next year?” (1 = *not well at all*; 7 = *very well*); (e) “How good would you be at learning something new in math?” (1 = *not at all good*; 7 = *very good; α* = *ω =* 0.89).

#### Parents’ Math Expectancy Beliefs (PEB)

To measure parents’ expectancy beliefs (PEB) in math, parents responded to a single item adapted from [29] measuring their perceived confidence of their children’s ability in math (e.g., Please indicate how much you agree with this statement. “My child will do very well with math activities”). The response of the scale item ranges from 1 (*strongly disagree)* to 7 (*strongly agree)*, where a higher score represents a greater confidence in their children’s math ability.

#### Adolescents’ Perceptions of Math Utility Value (AUV)

Three items were modified from [29] to assess adolescent’s perceptions of utility value (AUV) in math: (a) “How useful is what you learn in math?” (1 = *not at all useful*; 7 = *very useful*); (b) “For me, being good at math is…” (1 = *not at all important*; 7 = *very important*); (c) “Compared to other activities, how important is it to be good at math? (1 = *not at all important*; 7 = *very important; α* = 0.67; *ω* = 0.68).

#### Parents’ Perceptions of Math Utility Value (PUV)

Parents’ perceptions of math utility value was tapped by a single item adapted from [29]: “Please indicate how much you agree with this statement: Learning math will be useful for my child.” (1 = *not at all important*; 7 = *very important*).

#### MCSE career interest

Adolescents’ math intensive career interest (mathematics, computer science, statistics and engineering; MCSE) was measured by four items [adapted from 88,89], the sum was calculated in the current study. Example item: “In thinking about your future, how interested are you in possibly having mathematics/computer science/ statistics/engineering jobs?” (1 = *not all interested*; 6 = *very interested; α* = 0.81; *ω* = 0.80).

#### Controls

In this study, parent’s and adolescent’s gender (0 = *male*; 1 = *female*), ethnicity (0 = *Non-White*; 1 = *White*), age (in years), parent’s completed highest level of education (0 = *secondary school*; 1 = *college or undergraduate*; 2 = *master or PhD*) were assessed with self-reported items. Country (0 = *US or 1 = UK*) was also added as a control variable [37].

### Data analysis

**Fig 1:**
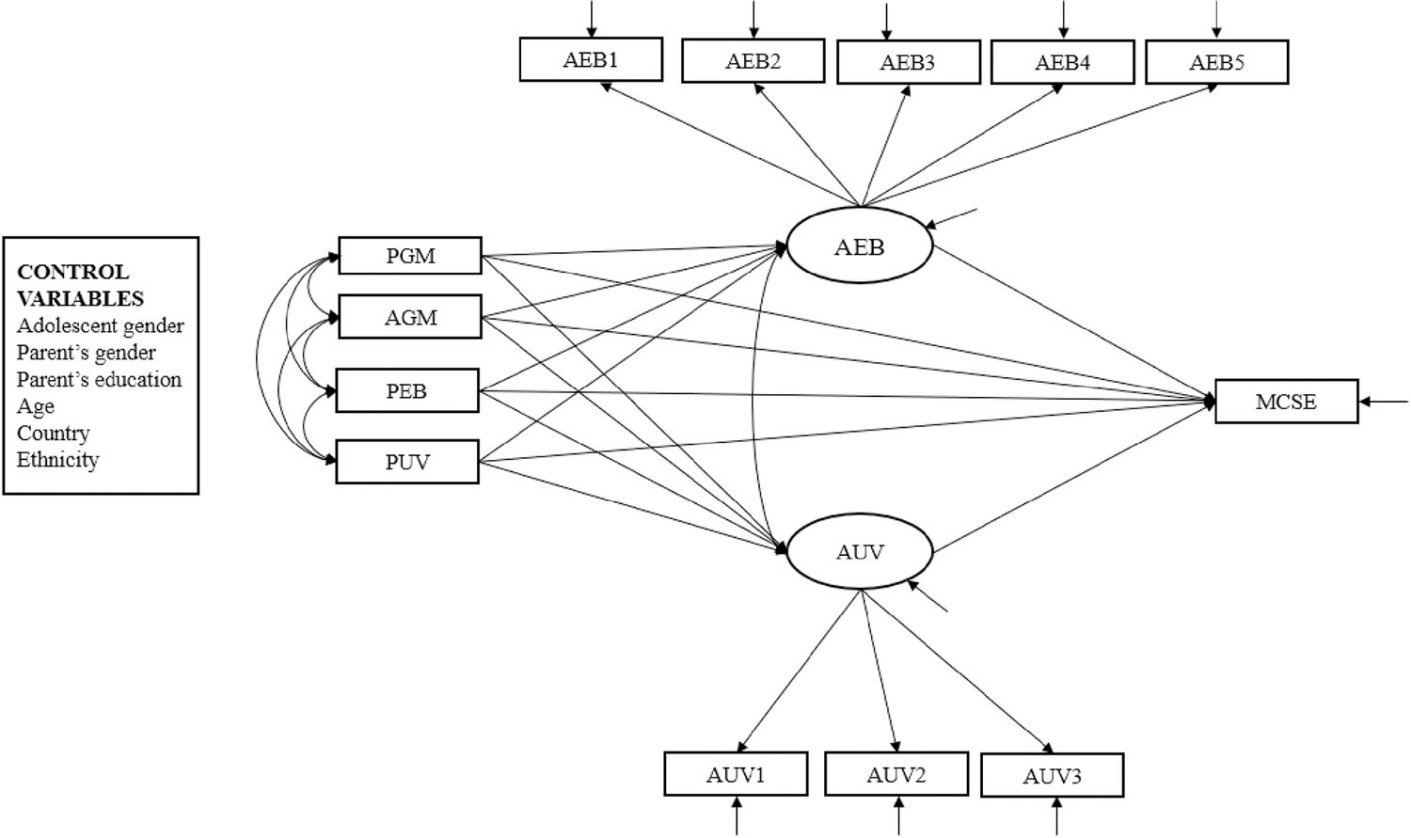
Conceptual model depicting the associations among PGM, AGM, AEB, AUV, PEB, PUV, and MCSE career interests. PGM = parent growth mindset; AGM = adolescent growth mindset; AUV = adolescent utility value; AEB = adolescent expectancy beliefs; PEB = parental expectancy beliefs, PUV = parental utility value; MCSE = math, computer science, statistics, and engineering career interests.

The present study utilizes structural equation modeling (SEM) analysis [90]. See Fig 1 for the hypothesized model of the current study. Before we analyzed the data, numerical tests of normality (i.e., skewness and kurtosis) and P-P plots were examined. All skewness (-1.65 to 0.082) and kurtosis (-0.813 to 2.76) values were in acceptable ranges (e.g., skewness: < 2; kurtosis: < 7) confirming normality at a univariate level [91]. To test the multicollinearity between independent variables, variance inflation factor (VIF) values were calculated. VIF values > 10 are not acceptable [90]. All VIF values are within acceptable ranges (1.05–1.15), which indicates that multicollinearity is not a problem at multivariate level. Spearman zero-order correlations were calculated for study variables using IBM SPSS Statistics 29.0 [92]. See Table 1.

**Table 1:** Descriptive statistics and spearman zero-order correlations among study variables.

Variable	1	2	3	4	5	6	7	8	9	10	11	12	13
1. Adolescent gender	1												
2. Parent’s gender	0.023	1											
3.Country	0.215**	-0.313**	1										
4. Parent’s education	-0.062	0.164*	-0.579**	1									
5. Adolescent’s ethnicity	-0.123*	0.188*	-0.581**	0.311**	1								
6. Adolescents’ age	0.087	-0.263**	0.471**	-0.349**	-0.179**	1							
7. PGM	0.116	-0.018	0.151	0.030	-0.033	-0.050	1						
8. AGM	0.00	-0.022	-0.054	0.097	0.090	-0.038	0.148	1					
9. AUV	-0.044	-0.132	0.055	-0.079	-0.091	-0.098	-0.038	0.078	1				
10.AEB	-0.064	-0.211**	-0.008	0.032	0.002	-0.170**	0.150	0.127*	0.288**	1			
11. PUV	0.054	0.071	-0.064	0.189*	0.130	-0.067	0.161	0.216**	0.228**	0.179*	1		
12.PEB	0.024	-0.146	-0.081	0.135	0.119	-0.116	0.133	0.151	0.126	0.473**	0.418**	1	
13. MCSE	-0.195**	-0.157*	-0.103	0.009	-0.015	-0.244**	0.043	0.186**	0.398**	0.427**	0.144	0.199*	1
*M*						15.20	5.58	5.48	17.06	28.06	6.61	5.90	12.66
SD						1.65	1.25	1.59	2.52	4.85	0.54	1.25	4.89
Skewness						-0.56	-1.49	-1.39	-0.90	-1.15	-0.95	-1.65	0.082
Kurtosis						0.224	2.66	1.47	1.31	1.32	-0.16	2.76	-0.813

*Note*. *M* = mean; SD = standard deviation; PGM = parent growth mindset; AGM = adolescent growth mindset; AUV = adolescent utility value; AEB = adolescent expectancy beliefs; PEB = parental expectancy beliefs; PUV = parental utility value; MCSE = math, computer science, statistics, engineering career interest.

* *p* < .05

** *p* < .01

*** *p* < .001.

#### Missing data analysis

The percentage of missing data was calculated by missing value analysis in IBM SPSS 29.0. [92]. The missing values varied from zero to 49.7% (see S1 Table for descriptive analysis). The pattern of missing data was tested with Little’s missing completely at random (MCAR) test [93]. The results indicated that the pattern of missing values was MCAR (see S2 Table for missingness patterns), χ^2^ = 80.405, *df* = 71, *p* = 0.208), which suggest that multiple imputation (MI) analysis is appropriate for the data [91]. If the percentage of the missingness is too large (e.g., from 40% to 80%), using MI is recommended practice [94] to correct the bias for variables where data are missing at random and MI more efficient than the complete case analysis [95]. MI with Bayesian analysis as described by [96] was carried out for all demographic and variables of interest in this study. Variables which did not include missing data (e.g., adolescents’ self-reported gender, country, ethnicity, SEB) were used as predictors and control variables in MI procedures. As previous studies suggested 3 or 5 imputations are sufficient [97]; and that using more than 10 imputations does not provide significant benefits [98], we used 10 imputed datasets in this study.

#### Model estimation and validation

Using SEVT [47], previous research [22] and Kline [90]’s model building approach the initial explanatory model for MCSE career interest was developed SEVT highlighted that parents’ characteristics (i.e., education, gender) and parents’ general and child specific motivational beliefs have a critical influence on adolescents’ career choices and academic outcomes [47]. Previous research established that parental beliefs [49,64,72], gender [99], education [100,101], and adolescents’ gender [22] are associated with adolescents’ motivational beliefs and interests in math and science. Therefore, parent and adolescent gender, parent education, age, and ethnicity were added as control variables to the hypothesized model. Adolescents’ expectancy beliefs and utility values in math were measured by latent variables.

The structural equations of the hypothesized model used in this study is as follows:

PGM = γ_1_ + γ_1_ *Gender*+ γ_1_*Pgen* + γ_1_*Pedu* + γ_1_*Age* + γ_1_*Country* + γ_1_*Ethnic* + ε_1_

AGM = γ_2_ + γ_2_*Gender*+ γ_2_*Pgen* + γ_2_*Pedu* + γ_2_*Age* + γ_2_*Country* + γ_2_*Ethnic* + ε_2_

PEB = γ_3_ + γ_3_*Gender*+ γ_3_*Pgen* + γ_3_*Pedu* + γ_3_*Age* + γ_3_*Country* + γ_3_*Ethnic* + ε_3_

PUV = γ_4_ + γ_4_*Gender*+ γ_4_*Pgen* + γ_4_*Pedu* + γ_4_*Age* + γ_4_*Country* + γ_4_*Ethnic* + ε_4_

AEB = β_0_+ β_1_*PGM* + β_2_*AGM* + β_3_*PEB*+ β_4_*PUV*+ γ_5_*Gender* + γ_5_*PGen* + γ_5_*Pedu* + γ_5_*Age*+ γ_5_*Country*+ γ_5_*Ethnic*+ ε_5_

AUV = β_5_+ β_5_*PGM* + β_6_*AGM* + β_7_*PEB*+ β_8_*PUV*+ γ_6_*Gender* + γ_6_*PGen* + γ_6_*Pedu* + γ_6_*Age*+ γ_6_*Country*+ γ_6_*Ethnic*+ ε_6_

MCSE = β_6_ + β_9_*PGM* + β_10_*AGM*+ β_11_*PEB*+ β_12_*PUV*+ β_13_*AEB* + β_14_*AUV*+ γ_7_*Gender* + γ_7_*PGen* + γ_*7*_*Pedu* + γ_7_*Age* + γ_7_*Country* + γ_7_*Ethnic* + ε_7_

Where, γ_1_, γ_2_, γ_3_, and γ_4_ represent the intercepts of endogenous observed variables: PGM, AGM, PEB, and PUV; the coefficients of β_0_ and β_5_ indicate the intercepts of endogenous latent variables: AEB and AUV, respectively. The intercept of MCSE career interest is represented by β_6._ The path coefficients represented by γ indicate the effect of exogenous variable; the path coefficients represented by *β* indicate the effect of endogenous (latent or observed) variables [102] and ε represents the random error term in the equation [e.g., 103]. For example, β_1_*PGM* is the effect of PGM (endogenous observed variable) on AEB (endogenous latent variable); γ_1_*Gender* is the effect of gender (observed exogenous variable) on PGM (observed endogenous variable). We used Baron and Kenny [104]’s approach to calculate the indirect effects between MCSE, PGM, AGM, PEB, and PUV via AEB and AUV, respectively.

Confirmatory factor analysis (CFA) was utilized to test the dimensionality of the latent factors using the fixed factor loading method [105]. The following statistics were used to evaluate the model fit: the model chi-square with degrees of freedom (*df*) and *p*-value, Steiger-Lind Root Mean Square Error of Approximation (RMSEA) with 90% CI, Bentler Comparative Fit Index (CFI), and Standardized Root Mean Square Residual (SRMR) [90]. The following values indicate a good fit on these indices: a non-significant chi-square test, CFI ≥ 0.95, RMSEA ≤ 0.06, and SRMR ≤ .08 [106]. CFA results confirmed adequate model fit for a single factor model of adolescents’ expectancy beliefs with five indicators (RMSEA = 0.068, 90% CI [0.013–0.119], CFI = 0.988, TLI = 0.975, SRMR = 0.030) and utility values in math with three indicators (RMSEA = 0.08, 90% CI [0.00–0.198], CFI = 0.969, TLI = 0.908, SRMR = 0.068). The structural model was tested with full information maximum likelihood (FIML) estimation using MPlus 8.4 statistical software [96].

## Results

Spearman zero correlation analysis and descriptive statistics are reported in Table 1. The model fit data indices for the SEM model across 10 imputed datasets suggested a good fit to the data (χ^2^ (85) = 131, 548, *p* = 0.009, RMSEA = 0.043, 90% CI [0.028–0.058], SRMR = 0.037, CFI = 0.95, TLI = 0.91). With a standardized solution, the proportion of explained variance (*R* square) for the latent variables are 0.33 (AEB) and 0.22 (AUV). The explained variance varies from 0.036 to 0.797 for observed outcome variables. Tables 2 and 3 present unstandardized and standardized direct and indirect effects with MCSE career interest.

**Table 2:** Estimates of path coefficients of the SEM model: Direct effects.

Effect	Path	Unstandardized estimate (SE)	Standardized Estimate	95% CI				
				LL	UL		*p*	
Direct effect on AEB								
	PGM → AEB	0.078 (0.054)	0.104	-0.029	0.185		0.154	
	AGM → AEB	0.012 (0.034)	0.019	-0.055	0.079		0.732	
	PUV → AEB	-0.036 (0.155)	-0.021	-0.340	0.268		0.805	
	PEB → AEB	0.337 (0.050)***	0.451***	0.238	0.435		*p* < .001	
Direct effect on AUV								
	PGM → AUV	-0.092 (0.052)	-0.179	-0.193	0.009		0.076	
	AGM → AUV	-0.005 (0.028)	-0.013	-0.060	0.049		0.847	
	PUV → AUV	0.410 (0.122)***	0.330***	0.172	0.648		*p* < .001	
	PEB → AUV	0.025 (0.046)	0.050	-0.065	0.115		0.576	
Direct effect on MCSE								
career interest	AEB → MCSE	1.321 (0.371)***	0.261***	0.594	2.048		*p* < .001	
	AUV → MCSE	2.307 (0.582)***	0.315***	1.167	3.448		*p* < .001	
	PGM → MCSE	-0.093 (0.279)	-0.024	-0.639	0.454		0.740	
	AGM → MCSE	0.352 (0.157)*	0.115*	0.045	0.659		0.024	
	PUV → MCSE	0.459 (0.689)	0.051	-0.892	1.810		0.499	
	PEB → MCSE	0.081 (0.302)	0.021	-0.512	0.673		0.794	

*Note*. PGM = parent growth mindset; AGM = adolescent growth mindset; AUV = adolescent utility value; AEB = adolescent expectancy beliefs; PEB = parental expectancy beliefs; PUV = parental utility value; MCSE = math, computer science, statistics, engineering career interest.; CI = confidence interval; LL = lower limit, UL = upper limit. Unstandardized CIs were reported.

* *p* < .05

** *p* < .01

*** *p* < .001.

**Table 3:** Estimates of path coefficients of the SEM model: Indirect effects, covariance/correlation.

Effect	Path	Unstandardized estimate (SE)	Standardized estimate	95% CI			
				LL	UL		*p*
Indirect effect of AEB							
	PGM → AEB → MCSE	0.105 (0.083)	0.028	-0.058		0.269	0.211
	AGM → AEB → MCSE	0.016 (0.046)	0.005	-0.074		0.105	0.728
	PUV → AEB → MCSE	-0.049 (0.217)	-0.006	-0.474		0.375	0.811
	PEB → AEB → MCSE	0.444 (0.136)***	0.117***	0.177		0.710	*p* < .001
Indirect effect of AUV							
	PGM → AUV → MCSE	-0.211 (0.124)	-0.056	-0.453		0.032	0.092
	AGM → AUV → MCSE	-0.012 (0.064)	-0.004	-0.138		0.113	0.847
	PUV → AUV → MCSE	0.953 (0.953)**	0.104**	0.218		1.687	0.009
	PEB → AUV → MCSE	0.057 (0.106)	0.015	-0.151		0.264	0.591
covariance/ correlation							
	AEB ↔ AUV	0.136 (0.047)***	0.292***	0.044		0.228	*p* < .001
	PGM ↔ AGM	0.140 (0.154)	0.074	-0.162		0.442	0.358
	PGM ↔ PEB	0.147 (0.122)	0.100	-0.092		0.385	0.220
	PGM ↔ PUV	0.073 (0.046)	0.118	-0.018		0.164	0.110
	PUV ↔ PEB	0.199 (0.057)***	0.319***	0.087		0.312	*p* < .001
	PUV ↔ AGM	0.108 (0.053)*	0.134*	0.005		0.211	0.034
	PEB ↔ AGM	0.182 (0.145)	0.097	-0.101		0.466	0.205

*Note*. PGM = parent growth mindset; AGM = adolescent growth mindset; AEB = adolescent expectancy beliefs; AUV = adolescent utility value; PEB = parental expectancy beliefs; PUV = parental utility value; MCSE = math, computer science, statistics, engineering career interest; CI = confidence interval; LL = lower limit, UL = upper limit. Unstandardized CIs were reported.

* *p* < .05

** *p* < .01

*** *p* < .001.

### The role of parent and adolescent growth mindset

The first research question was whether AGM was associated with AEB, AUV and MCSE career interest. AGM was weakly associated with MCSE career interest (*B* = 0.115, *p* < 0.05), but did not predict AEB (*B* = 0.019, *p* = 0.732) and AUV (*B* = -0.013, *p* = 0.847). The second research question was whether PGM predicts AEB, AUV and MCSE career interest. As shown in Table 2, PGM did not predict AEB (*B* = 0.104, *p* = 0.154), AUV (*B* = - 0.179, *p* = 0.076), or MCSE career interest (*B* = -0.024, *p* = 0.740).

### The role of parental motivational beliefs

The third research question examined the direct effects of parental motivational beliefs (PEB, PUV) on adolescent motivational beliefs (AEB, AEV) in math and MCSE career interest. As hypothesized, PEB predicted AEB (*B* = 0.451, *p* < 0.001); but did not predict AUV (*B* = 0.050, *p* = 0.576) and MCSE career interest (*B* = 0.021, *p* = 0.794). Further, PUV predicted AUV (*B* = 0.330, *p* < 0.001); but did not predict AEB (*B* = -0.021, *p* = 0.805) and MCSE career interest (*B* = 0.051, *p* = 0.499). The results suggest that if a parent had higher personal value of math and expectations from their children, then adolescents were more likely to have a higher personal value and expectations in math.

### The association between parental motivational beliefs and mcse career interest via adolescent motivational beliefs

**Fig 2:**
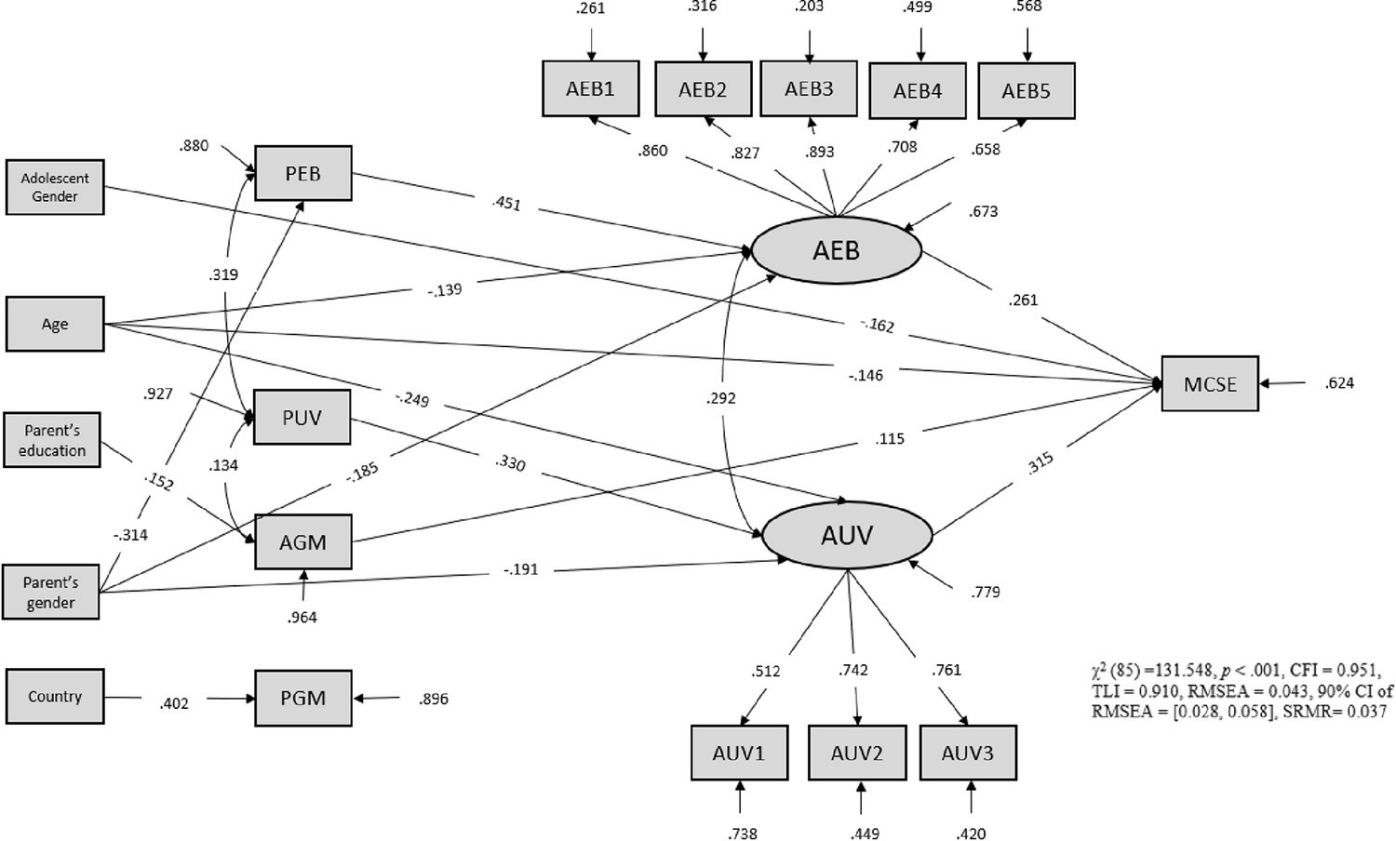
Standardized path coefficients for the model. PGM = parent growth mindset; AGM = adolescent growth mindset; AUV = adolescent utility value; AEB = adolescent expectancy beliefs; PEB = parent expectancy beliefs; PUV = parental utility value; MCSE = math, computer science, statistics, engineering career interest. Only significant paths are depicted.

The fourth question of this study concerned to what extent parental math motivational beliefs (PEB, PUV) relate to MCSE career interest via adolescent math motivational beliefs (AEB, AUV). As shown in Fig 2, the indirect link between PEB and adolescents’ MCSE career interest via AEB was found (*B* = 0.117, *p* < 0.001), suggesting that the relationship between parents’ math expectations and their adolescents’ higher interest in MCSE is accounted for by higher math expectation for adolescents. This result reveals that the association between PEB and higher career interest in MCSE fields was attributed to higher AEB in adolescence. Further analysis shows that PUV has an indirect positive effect on adolescents’ MCSE career interest via AUV (*B* = 0.104, *p* = 0.009). The results show that the association between parents’ personal value of math and their children’s higher career interest in MCSE was attributed to higher personal value in math for adolescents.

### The association between parent growth mindset and MCSE career interest via adolescent motivational beliefs

The fifth research question was to what extent math growth mindset (PGM, AGM) relates to MCSE career interest via adolescent motivational beliefs (AEB, AUV). The results show that the PGM did not predict MCSE career interest via AEB (*B* = 0.028, *p* = 0.211) and AUV (*B* = -0.056, *p* = 0.092). As can be seen in Table 3, AGM did not predict MCSE career interest via AEB (*B* = 0.005, *p* = 0.728) and AUV (*B* = -0.004, *p* = 0.847). As such, there was no evidence that parent and adolescent growth mindset had an indirect influence on adolescents’ MCSE career interests.

### Control variables

The effect of adolescents’ gender (0 = *male*; 1 = *female*) on MCSE career interest was negative and statistically significant (*B* = -0.162, *p* < 0.001). Age had a significant negative effect on AEB (*B* = -0.139, *p* < 0.05), AUV (*B* = -0.249, *p* < 0.01) and MCSE career interest (*B* = -0.146, *p* < 0.05). As shown in Fig 2, the effect of parents’ gender on PEB (*B* = -0.314, *p* < 0.001), AEB (*B* = -0.185, *p* = 0.006), and AUV (*B* = 0.191, *p* = 0.027) were negative and statistically significant. The effect of parent’s education level on AGM was positive and statistically significant (*B* = 0.152, *p* < 0.05). In addition, parents in the U.K. reported a greater growth mindset in math compared to their counterparts in the U.S. (*B* = 0.402, *p* < 0.001). Results indicated that ethnicity had no significant effect on the variables of interest.

## Discussion

Thus far, previous studies have identified links between mindset, motivational beliefs in math [57,107], and STEM career outcomes [22]. However, there are few studies focusing on mindset in a family context [25], and in particular in informal STEM learning settings [36,37]. Our findings extend SEVT [47] by including on domain specific parent math mindset, motivational beliefs and math career orientations in a sample of teens who joined a STEM youth program in the U.S. and the U.K. Our novel findings document important ways in which parent growth mindset and motivational beliefs as well as adolescents’ own attitudes and beliefs shape math intensive career interest.

The current study sought to examine whether parent and adolescent growth mindset, and motivational beliefs in math would predict career interest in MCSE. First, this study found that adolescent math expectancy beliefs [30] and perceptions of math utility values [22,108] play crucial roles in their career interests in MCSE suggesting greater likelihood of pursuing a math career controlling for student gender, age, parent gender, parent highest completed education level and ethnicity. In contrast to earlier findings [22], we found that adolescent math growth mindset weakly predicted MCSE career interest. More importantly, we found that parental expectancy beliefs predicted adolescents’ MCSE career interests through adolescents’ expectancy beliefs in math. The results of the present study support SEVT theory and demonsrate that parental perceived value of math predicted adolescents’ MCSE career interest through adolescents’ perceived value of math. Below we discuss implications of the findings for each research question and directions for future research.

### Growth mindset, motivational beliefs and MCSE career interest

The first research question in this study sought to determine whether adolescent math growth mindset would predict their math motivational beliefs (e.g., expectancy beliefs and utility values) and MCSE career interest. Contrary to expectations, this study did not find a significant association between adolescent growth mindset and their motivational beliefs. These results reflect those of Degol et al. [22], who found non-significant associations between student math growth mindset and math expectancy beliefs. These results may be explained by the dynamic association between growth mindset and motivational beliefs [109]. It may be that as adolescents strengthen their growth mindset, they also become motivated and vice-versa. Reciprocal relationships in across-lagged research design might provide more detailed information about the developmental trajectories and patterns of the relationship between growth mindset and motivational beliefs [22].

Another important finding is that adolescent growth mindset weakly predicts their MCSE career interest, demonstrating that the higher an adolescent’s growth mindset in math is, the more they have career interests in MCSE. The weak association between adolescent math growth mindset and MCSE career interest might be explained by a potential loss in predictive power of student mindset on long term career outcomes in STEM. Prior findings for mindset show that effect sizes are often small [110]. Our power analysis indicated that our study was appropriately powered for small effect sizes. This finding was also reported by Cheng et al. [28], who found that the association between student math growth mindset and career plans was stronger in the hard sciences (e.g. engineering, math) compared to soft sciences (e.g., architecture, health) in STEM domains for adolescents. Consistent with the previous studies, our findings suggest the importance of promoting math growth mindset for adolescents.

On the second question, consistent with previous research [111], parent growth mindset did not predict MCSE career interest in this study. Parent growth mindset also did not predict adolescents’ motivational beliefs in math. This finding is contrary to that of Cheng and his colleagues [28] who found significant associations between parent growth mindset in math and their adolescent’s perceived ability in math. A possible explanation for this might be the discrepancy between self-reported parent and adolescent growth mindsets. Adolescents’ perceptions of their parents’ mindset might be different from their parents’ self-reported mindset [25]. Prior research has often noted the limitations of asking children to report on their perceptions of parental behaviors, such as parental rejection [112], but only gathering parent-report may also pose challenges. Future research should aim to examine both parent report of their mindset as well as adolescent report of their perception of their parent mindset to more carefully explore potential differences. These findings draw our attention to the importance of explicit parent-adolescent communication in shaping adolescents’ perceptions of mindset in a family context.

With respect to the third research question, we found that parental expectancy beliefs and utility values in math predicted adolescents’ math expectancy beliefs and task utility values, respectively. Consistent with the previous studies [38,113], our findings suggest that parents with high expectations for their adolescents and who value math highly might support their adolescents in math-related tasks in informal learning settings (e.g., visiting museums, math-related discussions, etc.) which may help to increase their adolescent’s math motivation. Surprisingly, the effects of parental expectancy beliefs and utility values in math on MCSE career interest are not statistically significant. A possible explanation for these results may be the differences between parents’ self-reported motivational beliefs and adolescents’ perceptions and interpretations of their parents’ motivational beliefs [38,83]. A further study with more focus on adolescents’ perceptions about their parents’ beliefs and values is therefore suggested.

### The links between parental motivational beliefs and MCSE career interests via adolescents’ motivational beliefs

The fourth research question sought to determine whether the indirect links between parental math motivational beliefs and MCSE career interest are predicted by adolescents’ math motivational beliefs. As hypothesized, an indirect link was found between parental expectancy beliefs and MCSE career interest via adolescents’ expectancy beliefs. This study also confirms the indirect link between parental perceptions of math importance and MCSE career interest via adolescents’ perceptions of math importance. These results provide further support for the work of other studies linking parental, and youth shared motivational beliefs and math career orientation [26,77]. These findings highlight the important ways in which parents can shape their adolescents’ career trajectories. Their expectancies foster adolescent’s own expectations which have carry-on effects on adolescents’ career interests. Thus, parents should attend carefully to the messages they communicate about different fields of study and their beliefs about those fields.

### The links between growth mindset and MCSE career interests via adolescents’ motivational beliefs

With respect to the fifth research question, we studied the indirect associations between parent and adolescent math growth mindset and MCSE career interest via adolescents’ motivational beliefs. We were unable to demonstrate any indirect effects of parents’ and adolescents’ growth mindset on MCSE career interest. This result may be explained by the differences in perceived and self-reported parent mindsets [25,28,85]. Adolescents might have difficulties observing and interpreting their parents’ mindsets [85]. In order to improve accuracy of adolescents’ perceived parental perceptions, parental value communication, family value agreement, and parenting styles need to be taken into account [114]. Adolescents receive messages both directly and indirectly from parents and other parental factors may be more important in shaping adolescents’ motivation than growth mindset.

### Limitations and implications of the present study

Although our findings provide evidence to support a significant connection between parent factors and adolescents’ math-related career interests, we did not explore how parents convey their beliefs to their children in this study. Parents play a critical role in shaping adolescents’ career interests and choices in STEM [115,116]. Research with adolescents on math-intensive careers suggest that parents had a strong influence on African—American students’ engineering career choices [117]. Godwin and her colleagues [84] reported that having a father who is an engineer is a negative predictor of engineering career choice; however, other familial engineering figures are positive predictors of engineering career choice. Previous research has also established that specific parental behaviors (e.g., psychosocial support, career action) and parent-adolescent relationships predict young adolescents’ career decisions and development [118]. For example, Keller and Whiston [118 p. 211] found that “young adolescents need to know their parents are interested in them as individuals, believe in their abilities, trust them to make good decisions, and are proud of them”. They also reported that the discrepancy between parents’ and adolescents’ perceptions of family relationships had a significant negative effect on adolescent’s career decision-making self-efficacy. There is abundant room for further progress in determining associations between parent-adolescents’ relationship, parent behavior and attitudes and MCSE career interest and choices.

Further research should be undertaken to explore how particular parental influences [84] and family dynamics (e.g., father-child interaction, siblings mindset) [25] are exerted in adolescents’ math career orientations. The present study has been one of the first attempts to thoroughly examine the parent factors in an informal STEM learning context. While it is a strength to focus on adolescents engaged in informal STEM learning, research should replicate these findings with samples of adolescents not participating in informal STEM programs. Further studies regarding the role of informal education organizations and practitioners on parent factors (i.e., supportive behaviors) would be worthwhile [83].

The findings of this study have significant implications for the understanding of the role of parent factors on adolescents’ motivational beliefs and math career orientations. Our results support strong recommendations for parents to encourage their adolescents’ motivation and interest in math by using more explicit about communication in their conversations and parenting style with their adolescents. The findings of this research also suggest that informal STEM youth programs might provide a valuable source for parents to support their adolescents’ math career interest through increasing shared motivational beliefs.

Consistent with previous findings [22,44,119], adolescent gender had a negative effect on MCSE career interest, indicating females would be less likely to pursue careers in MCSE. The effect of age on adolescent motivational beliefs and MCSE career interest was negative, suggesting adolescents were less likely to pursue math-intensive careers at a young age [120]. Previous studies have demonstrated that there are differences between parental beliefs and student academic outcomes in particular STEM domains [26]. Parents’ gender stereotypes have a direct effect on their daughters perceived math abilities [113,121] and parents tend to underestimate girls’ math abilities [26,113]. We found that parents’ gender had a negative effect on adolescents’ motivational beliefs and parental expectancy beliefs in math. These results suggested that mothers more likely would have lower expectations for their children in math. However, this finding must be interpreted with caution because the sample characteristics may be somewhat limited by the parents’ gender in this study. Approximately 87% of the parent sample consists of mothers and 69.3% of adolescents were female adolescents in this study. Therefore, future studies should focus more on external replications with larger samples and equal representations of each gender group to reach more generalizable conclusions and improve the external validity of the findings. Parent education level had a positive effect on adolescents’ growth mindset. Although we did not expect significant differences in self-reported parent growth mindset in two countries, it is interesting that parents from the U.K. reported higher scores for growth mindset compared to parents from the U.S. Further research could explore how the influence of mindset in family context on motivational beliefs differs in multicultural learning settings.

Adolescents’ math motivational beliefs are predicted by parental math motivational beliefs. However, perceived parent factors (e.g., motivational beliefs, parent mindset) may have a greater influence than self-reported parent factors [38]. In addition, it seems possible that parents’ supportive behaviors might mediate the associations between shared motivational beliefs [72] and adolescents’ math career orientation. Therefore, further research needs to examine more closely the links between perceived parent factors and parent behaviors (i.e., role modeling, co-activity, encouragement), and math outcomes in adolescence. Also, the data presented here were limited to self-report measures. Future research should more fully evaluate parent factors from adolescents’ perspectives.

The majority of the sample consists of white female adolescents and their mothers. Thus, the current study is limited by the relatively small sample of male adolescents. Given that female adolescents are under-represented in MCSE science fields, we believe it is a strength that females are overrepresented in the current study. However, future studies should aim to replicate our findings with a more gender-balanced sample to ensure that the patterns hold for both male and female adolescents. Also, given that the sample is predominantly White girls and their mothers, the findings may not be generalizable to girls from other ethnic/racial backgrounds and the findings may not be applicable to fathers, thus, future research should aim to focus even more directly on the experiences of ethnically marginalized adolescents and father reports.

Although previous studies highlighted the critical influence of mothers’ beliefs on their children’s perceived math ability [49], with a small sample size, caution must be applied, as the findings might not be transferable to variety of family context (e.g., fathers, siblings). Given the dynamic features of mindset and career interest, it would be important to explore the reciprocal longitudinal relations of parent factors, mindset, and math career orientations [22]. Previous studied reported that parents’ socioeconomic status [122,123] and occupation [84,124] are strongly linked to adolescent’s career orientations. It is also recommended that further research be undertaken on the potential confounding variables including parents’ socioeconomic status and occupation.

Finally, the lack of socioeconomic data in this study adds further caution regarding the generalizability of these findings. Socioeconomic status (SES) has been shown to have significant effects on math intensive career choices. For example, Potvin and her colleagues [125] found that engineering students had lower socioeconomic backgrounds (assessed by parents’ highest education level, *p* < 0.05) compared to science students. They reported that students with strong math skills who come from lower SES background are encouraged to choose highly paying math intensive careers such as engineering with a pragmatic and materialistic motivation [125]. Further studies, which take socioeconomic variables into account, will need to be undertaken.

## Conclusion

The results of this study extended SEVT by including domain-specific parental motivational beliefs, growth mindset and adolescents’ math intensive career interest in a unique sample of volunteer teens who joined a STEM youth program in the U.K. and the U.S. using SEM analysis. Our results support previous findings about the critical role of parents’ expectancy beliefs and utility values [63,83] on adolescents’ motivational beliefs and their math career orientation. Our findings indicate that adolescent math growth mindset weakly predicted their career interests in math intensive domains. Overall, the results have shown that parental perceptions of their children’s math ability and their personal value of math regulate their adolescents’ self-perceived math ability and perceived value of math and may contribute to math-intensive career interest indirectly through adolescents’ motivational beliefs.

## Supporting information

S1 Table Descriptive analysis of missing data. (DOCX)

S2 Table Missingness patterns. (DOCX)

S1 File (DOCX)
