# Supplementary material for: The relations between growth mindset, motivational beliefs, and career interest in math intensive fields in informal STEM youth programs
Source: PLoS One. 2024 Apr 9;19(4):e0294276. doi: 10.1371/journal.pone.0294276 (PMC11003616; doi:10.1371/journal.pone.0294276)
Supplement: S1 Table — (DOCX) [file pone.0294276.s001.docx]

**S1 Table. Descriptive Analysis of Missing Data (*n* = 290)**

| Variable | N of missingness | % Missing | Mean | *SD* | Scale |
| --- | --- | --- | --- | --- | --- |
| PGM | 144 | 49.7 | 5.58 | 1.25 | Numerical (1 to 7) |
| AGM | 7 | 2.4 | 5.48 | 1.59 | Numerical (1 to 7) |
| PEB | 132 | 45.5 | 5.90 | 1.25 | Numerical (1 to 7) |
| PUV | 133 | 45.9 | 6.61 | 0.54 | Numerical (1 to 7) |
| AEB1 | 0 | 0 | 5.85 | 1.12 | Numerical (1 to 7) |
| AEB2 | 1 | 0.3 | 5.24 | 0.91 | Numerical (1 to 7) |
| AEB3 | 0 | 0 | 5.33 | 1.52 | Numerical (1 to 7) |
| AEB4 | 4 | 1.4 | 5.97 | 1.05 | Numerical (1 to 7) |
| AEB5 | 0 | 0 | 5.77 | 1.06 | Numerical (1 to 7) |
| AUV1 | 1 | 0.3 | 5.26 | 1.29 | Numerical (1 to 7) |
| AUV2 | 0 | 0 | 6.10 | 0.88 | Numerical (1 to 7) |
| AUV3 | 0 | 0 | 5.71 | 1.01 | Numerical (1 to 7) |
| MCSE - MATH | 0 | 0 | 3.37 | 1.58 | Numerical (1 to 6) |
| MCSE - CS | 2 | 0.7 | 2.71 | 1.38 | Numerical (1 to 6) |
| MCSE -STATISTICS | 1 | 0.3 | 3.39 | 1.58 | Numerical (1 to 6) |
| MCSE - ENGINEER | 0 | 0 |  |  | Numerical (1 to 6) |
| AGE | 9 | 3.1 | 15.20 | 1.65 | Numerical (10 to 20) |
| GENDER | 0 | 0 | 3.17 | 1.56 | 0 = Male, 1 = Female |
| P_GEN | 128 | 44.1 | - | - | 0 = Male, 1 = Female |
| P_EDU | 138 | 47.6 | - | - | 0 = Secondary school,  1 = College or undergraduate,  2 = Master or PhD |
| COUNTRY | 0 | 0 | - | - | 0 = the U.S.,  1 = the U.K. |
| ETHNICITY | 0 | 0 | - | - | 0 = non-White,  1 = White |

*Note.* PGM = parent growth mindset; AGM = adolescent growth mindset; PEB = parent expectancy beliefs; PUV = parent utility value; AEB = adolescent expectancy beliefs; AUV = adolescent utility value; MCSE = math, computer science, statistics, and engineering career interest, CS = computer science, P_GEN = parent’s gender; P_EDU = parent’s highest completed education. SD = standard deviation
